# Isolated Displaced Chaput Fracture (Rammelt Type III) With Significant Intra-articular Displacement: A Case Report

**DOI:** 10.7759/cureus.114421

**Published:** 2026-08-12

**Authors:** Laith Hseinat, Majdala Al-Bataineh, Hamza A Abuain, Osama Abu Zaid, Murad Abu Jalboush, Yazan Al Omari, Shujaa Al-Jarajreh, Alaa Tawalbeh

**Affiliations:** 1 Department of Orthopaedics, Jordanian Royal Medical Services, Amman, JOR; 2 Faculty of Medicine, Yarmouk University, Irbid, JOR; 3 Department of Orthopaedic Surgery, Jordanian Royal Medical Services, Amman, JOR

**Keywords:** ankle fracture, anterior-inferior tibiofibular ligament (aitfl), chaput fracture, orif, syndesmotic injury

## Abstract

The distal tibiofibular syndesmosis, which is essential for preserving ankle stability, is often involved in ankle injuries. Avulsion fractures of the anterior inferior tibiofibular ligament, also referred to as Chaput fractures, if left untreated, may go undetected on plain radiographs and result in chronic instability. For precise evaluation and treatment planning, advanced imaging techniques such as computed tomography (CT) are frequently necessary. We present the case of a 23-year-old man who fell from a height of 2 m and suffered a twisting injury to his right ankle and was seen at King Hussein Medical Hospital. A Chaput fracture was suggested on the first radiographs. A single displaced Chaput fracture with an articular step-off larger than 2 mm was discovered after additional CT assessment. An anterolateral approach to the ankle was used to access the fracture and to perform internal fixation. A progressive physiotherapy rehabilitation protocol followed his unremarkable operative fixation. The patient regained complete range of motion with no pain or functional limitations at the three-month follow-up. Ankle fractures are frequently linked to syndesmotic injuries, which can be challenging to diagnose with just plain radiographs. CT imaging enhances the identification of avulsion fragments and offers a more accurate assessment of the displacement and shape of fractures. Syndesmotic stability may be attained without further fixation by simply fixing the Chaput fragment. Chaput fractures must be accurately diagnosed and treated to restore ankle stability and avoid long-term problems. Anatomical reduction may reduce the need for extra syndesmotic fixation in certain individuals, and CT imaging is an important part of the evaluation process.

## Introduction

Ankle fractures are among the most common musculoskeletal injuries encountered in orthopedic practice and represent a substantial proportion of emergency department presentations in adults. They affect individuals across all age groups, although their incidence is particularly high among athletes and is increasing in the elderly population. Despite advances in diagnosis and treatment, ankle fractures remain associated with significant morbidity, as a considerable proportion of patients develop post-traumatic osteoarthritis secondary to residual joint incongruity or unrecognized associated injuries. Missed syndesmotic injuries and overlooked fracture components may result in chronic ankle instability, persistent pain, and progressive joint degeneration, emphasizing the importance of accurate diagnosis and appropriate management [1].

The ankle is a highly congruent and biomechanically complex joint that depends on the coordinated interaction of its osseous architecture, ligamentous stabilizers, and surrounding musculature to maintain stability during weight-bearing and rotational movements. The distal tibiofibular syndesmosis forms an integral component of the ankle mortise and consists of the anterior inferior tibiofibular ligament (AITFL), posterior inferior tibiofibular ligament (PITFL), interosseous ligament, and transverse tibiofibular ligament. Together, these structures stabilize the distal fibula within the tibial incisura while allowing the physiological micromotion necessary for normal ankle function. Injuries to this complex may present as ligamentous disruption or avulsion fractures at the ligament insertion sites and can significantly compromise ankle stability if left untreated [2,3].

Among the syndesmotic stabilizers, the AITFL serves as one of the principal restraints against external rotational forces and is particularly susceptible to injury. Avulsion fractures at its tibial insertion are referred to as Chaput fractures. While syndesmotic injuries are frequently encountered in association with ankle fractures, isolated syndesmotic injuries without accompanying malleolar fractures are uncommon [4]. Failure to recognize syndesmotic disruption may result in widening of the ankle mortise, talar malalignment, persistent instability, chronic pain, and progressive post-traumatic degenerative changes. Moreover, small avulsion fragments, such as Chaput fractures, may be easily overlooked on conventional radiographs, increasing the risk of delayed diagnosis and inferior functional outcomes [5].

Chaput fractures are typically caused by an external rotational or twisting mechanism of the ankle and represent avulsion injuries of the anterolateral distal tibia at the insertion of the AITFL. Although these injuries are well recognized in adolescents as the osseous equivalent of a Tillaux fracture, isolated Chaput fractures in skeletally mature adults remain distinctly uncommon, as ligamentous rupture is generally more frequent than osseous avulsion in this age group [6]. Most adult Chaput fractures occur in conjunction with malleolar fractures or more complex ankle injury patterns, whereas isolated displaced Chaput fractures have been only rarely described. Consequently, these injuries may be underrecognized, particularly by clinicians who are unfamiliar with this uncommon fracture pattern.

Because of the complex three-dimensional anatomy of the ankle, conventional radiographs may underestimate fracture displacement, articular involvement, and fragment morphology. Computed tomography (CT), particularly when combined with three-dimensional reconstruction, provides superior visualization of fracture configuration and displacement, facilitating accurate diagnosis and preoperative planning [7]. Management depends primarily on fracture displacement and restoration of joint congruity. Non-displaced fractures may be managed conservatively, whereas displaced fractures, particularly those with an articular step-off exceeding 2 mm, generally require anatomical reduction and internal fixation to restore joint congruity, maintain syndesmotic stability, and minimize the risk of long-term complications [1,2,8].

Anterior malleolar fractures, including Chaput fractures, have been classified by Rammelt et al. into three morphological patterns based on fracture configuration and articular involvement. This classification assists in understanding injury severity, guides surgical planning, and helps determine the most appropriate fixation strategy [8]. Anatomical reduction remains the primary goal of treatment, and fixation of the avulsed fragment alone may restore syndesmotic stability in selected patients, potentially eliminating the need for additional syndesmotic fixation. Nevertheless, the optimal management strategy remains controversial and requires careful preoperative assessment and intraoperative evaluation [3].

Unlike osteoarthritis affecting other major lower-extremity joints, ankle osteoarthritis is predominantly post-traumatic, most commonly developing after ankle fractures, ligamentous injuries, or fractures involving the distal tibia or talus [9]. Given the rarity of isolated displaced Chaput fractures in adults and the limited literature describing their diagnosis and surgical management, this case highlights the importance of maintaining a high index of suspicion in patients presenting with rotational ankle injuries. It further emphasizes the value of CT imaging for accurate fracture characterization and surgical planning, as well as the role of anatomical reduction in restoring syndesmotic stability without the need for additional syndesmotic fixation.

## Case presentation

In January 2026, a 23-year-old male presented to the emergency department two hours after sustaining a twisting injury to his right ankle following a fall from a height of approximately 2 m. He complained of severe right ankle pain and an inability to bear weight immediately after the injury.

On initial assessment, the patient was conscious, alert, and oriented, with stable vital signs. Clinical examination of the right ankle revealed moderate anterolateral swelling, localized tenderness over the anterolateral distal tibia and syndesmotic region, and painful restriction of both active and passive ankle movements. There was no gross deformity, skin compromise, open wound, or fracture blisters, and the compartments were soft without clinical evidence of compartment syndrome. Distal neurovascular examination was unremarkable, with intact motor and sensory function, palpable dorsalis pedis and posterior tibial pulses, and normal capillary refill.

**Figure 1:**
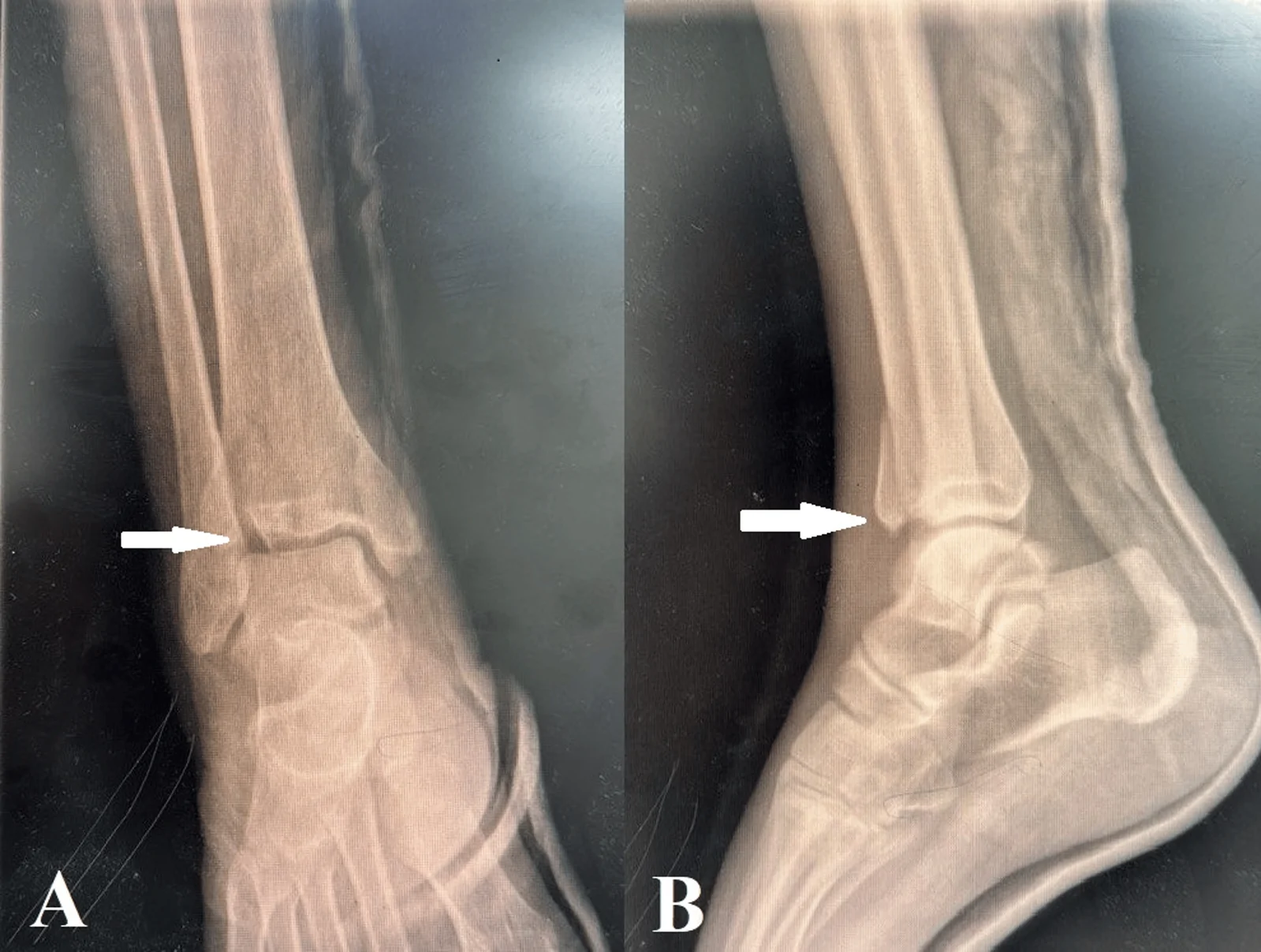
Initial X-rays. Initial presentation with anteroposterior (A) and lateral (B) X-ray views showing an isolated displaced Chaput fracture (arrow).

**Figure 2:**
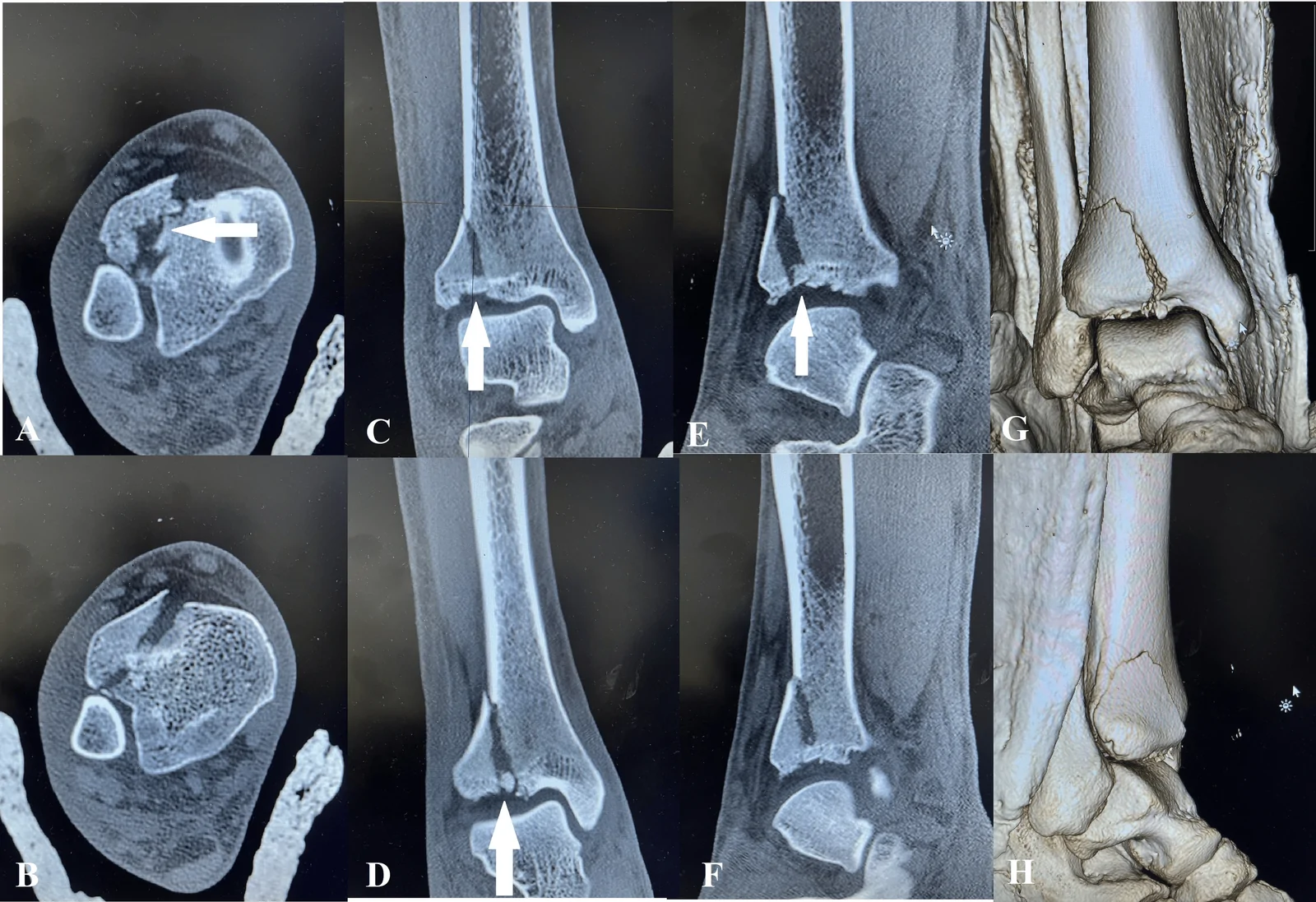
Initial CT scan. (A,B) Axial, (C, D) coronal, (E, F) sagittal, and (G, H) three-dimensional reconstruction CT scan cuts showing an isolated Chaput fracture with displacement greater than 2 mm and an associated articular step-off with incarcerated articular fracture piece (arrow).

**Figure 3:**
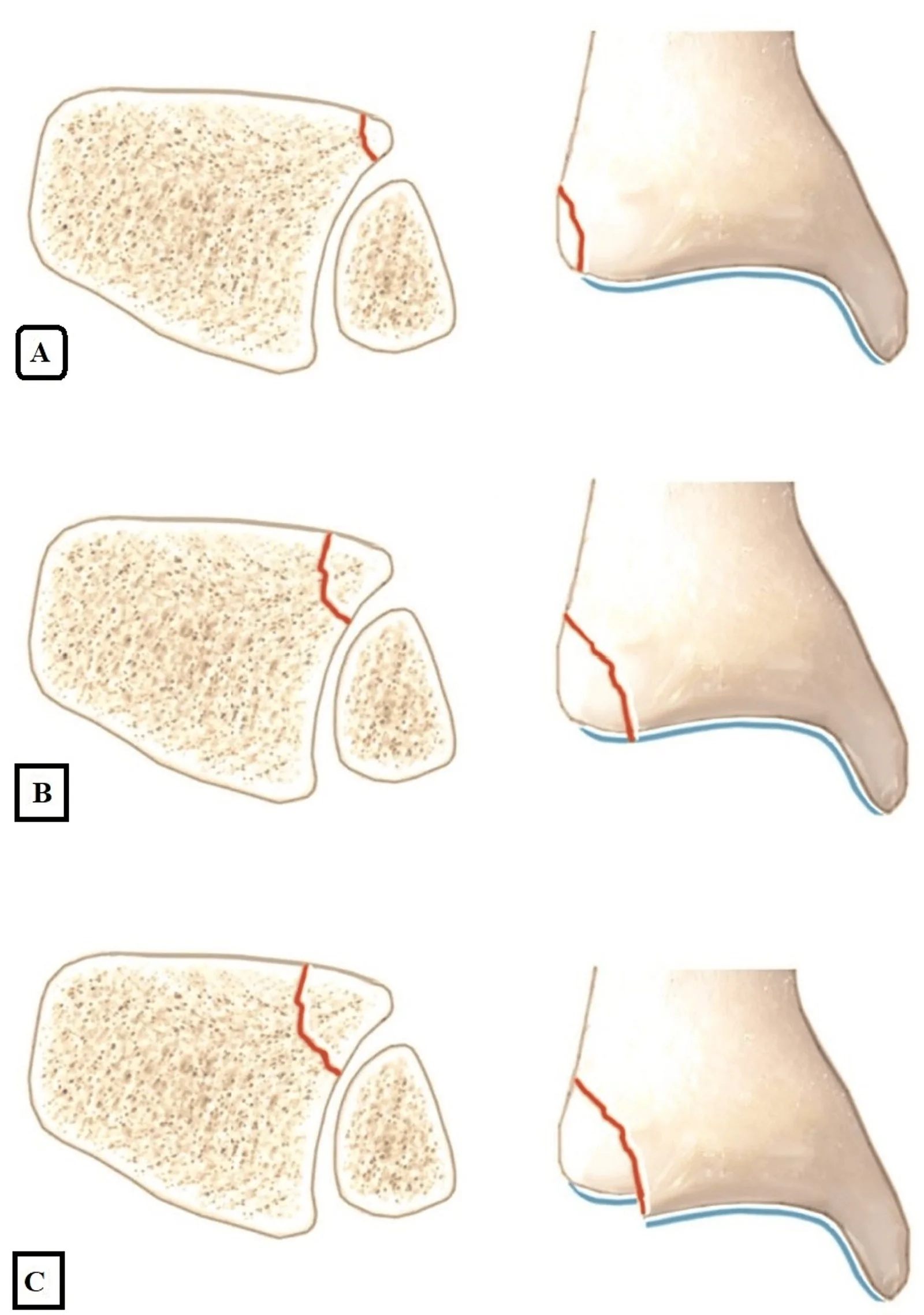
The Rammelt classification for Tillux-Chaput fractures. (A) Type 1: extra-articular avulsion. (B) Type 2: fracture with involvement of the joint and incisura. (C) Type 3: fracture with impaction of the anterplateral tibial plafond. Reproduced with permission from Springer Nature (Rammelt et al. [10]).

Plain radiographs of the right ankle demonstrated a displaced isolated Chaput fracture (Figure 1). A below-knee posterior back slab was applied, and the patient was admitted for further evaluation and soft-tissue management. Subsequent CT of the ankle confirmed an isolated Chaput fracture with more than 2 mm of displacement, an intra-articular step-off, and an incarcerated osteochondral fragment within the ankle joint (Figure 2). Based on the fracture morphology, the injury was classified as Rammelt type III (Figure 3). Owing to the degree of displacement and articular involvement, operative fixation was planned once the soft-tissue swelling had sufficiently subsided.

**Figure 4:**
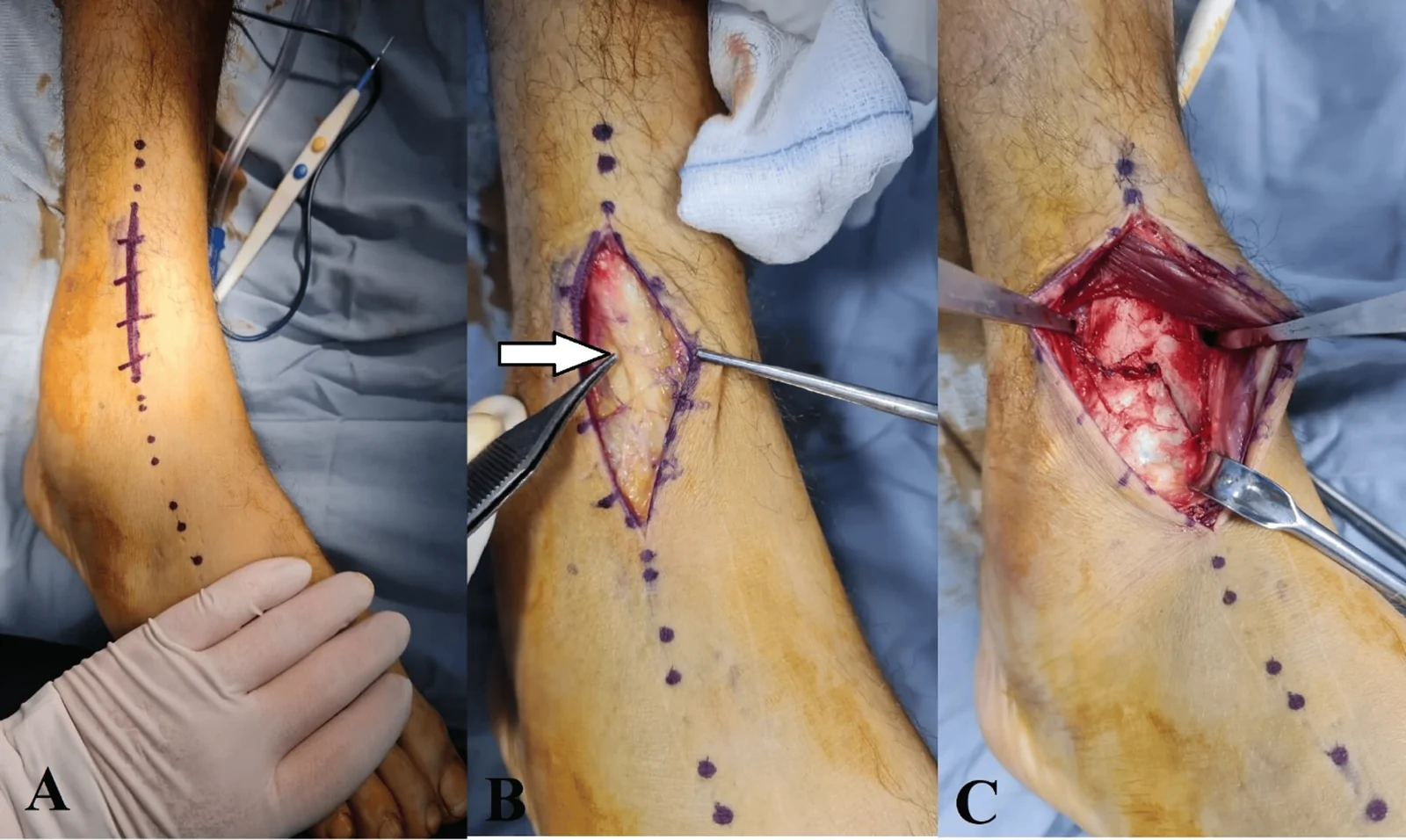
Clinical intraoperative images. (A) Clinical intraoperative image showing the anterolateral approach with a slightly curved incision made between the tibia and fibula. It begins approximately 5 cm proximal to the joint and extends distally toward the fourth metatarsal. (B) Clinical intraoperative image showing a superficial dissection of the superficial peroneal nerve (arrow). (C) Clinical image showing the displaced fracture after incising the joint capsule.

**Figure 5:**
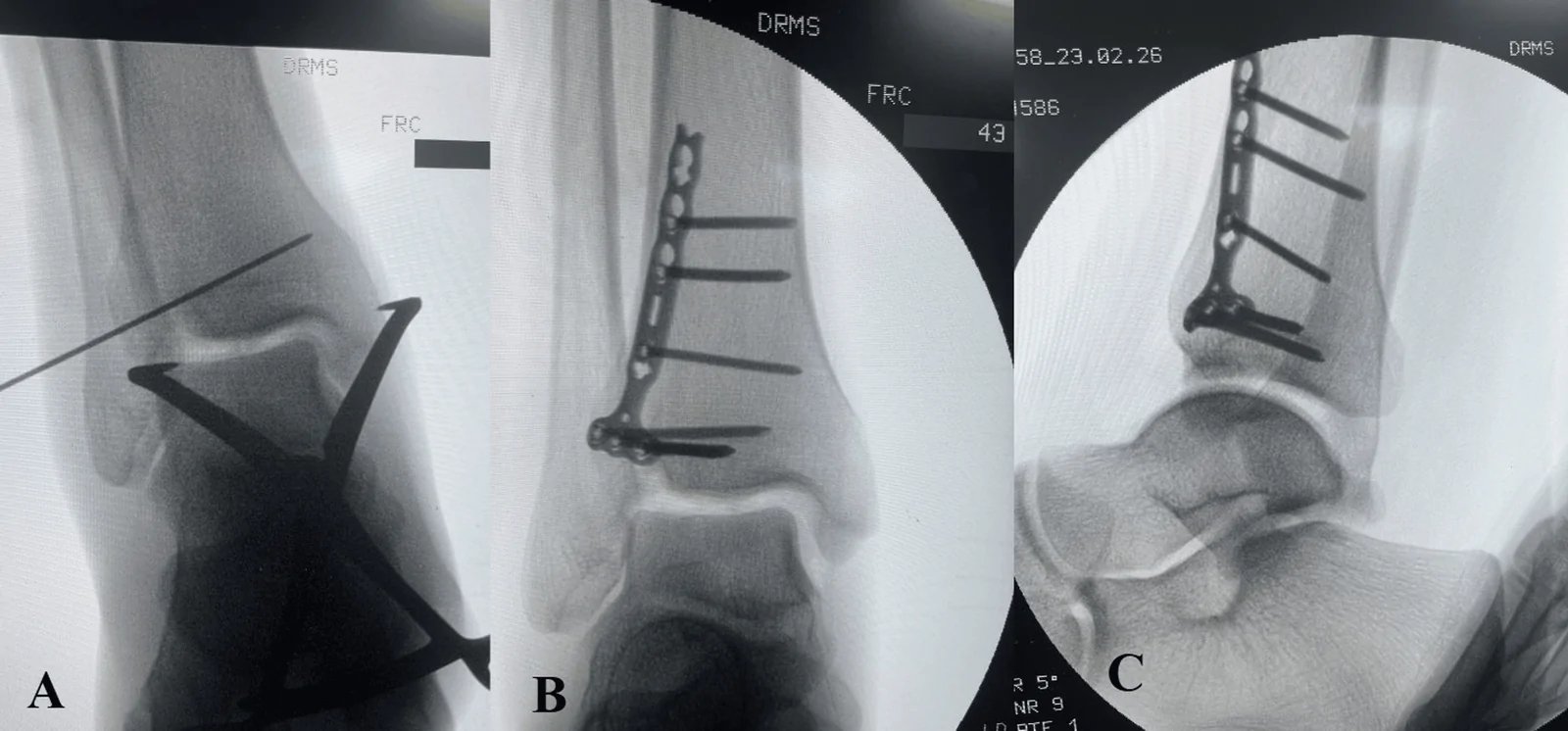
Intraoperative fluoroscopic images. Intraoperative fluoroscopic images showing excellent reduction of the Chaput fragment and restoration of the congruency of the articular surface. (A) Anteroposterior view of the ankle joint showing the provisional fixation of the reduced Chaput fragment by the C-clamp and the K-wire. (B, C) Ankle anteroposterior (B) and lateral (C) views showing the definitive fixation using the 2.7 mm T-plate with screws.

On the fifth day after admission, following resolution of the soft-tissue swelling, the patient underwent open reduction and internal fixation under general anesthesia with tourniquet control in the supine position. An anterolateral approach to the ankle was utilized, with careful identification and protection of the superficial peroneal nerve. Following a Z-shaped capsulotomy, the Chaput fragment was mobilized using an open-book technique, allowing visualization of the incarcerated osteochondral fragment. The incarcerated fragment was anatomically reduced, followed by reduction of the Chaput fragment to its native position (Figure 4). Reduction was temporarily maintained using a pointed reduction clamp and provisional Kirschner-wire fixation before definitive fixation with a 2.7-mm locking compression T-plate and screws under fluoroscopic guidance (Figure 5).

**Figure 6:**
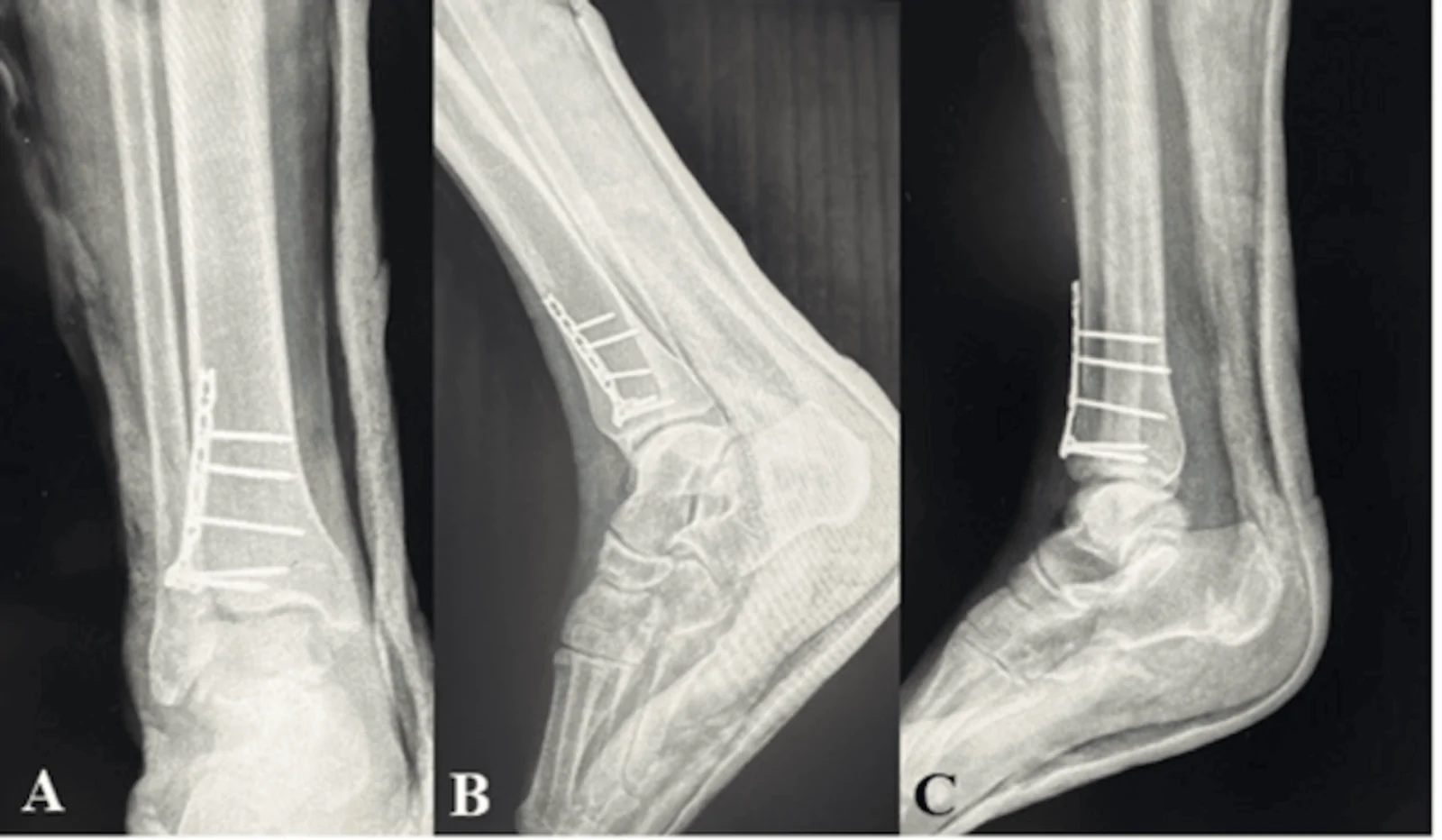
Initial ankle postoperative X-rays. Initial ankle postoperative anteroposterior (A), lateral (B) and external rotation (C) X-ray views showing the excellent reduction of the Chaput fragment, the final restoration of the joint congruency and alignment, and the final position of the plate.

Following definitive fixation, syndesmotic stability was assessed intraoperatively using both the external rotation stress test and the Cotton test under fluoroscopic guidance. No tibiofibular diastasis, medial clear space widening, or residual syndesmotic instability was observed. Therefore, additional syndesmotic fixation was not required. A below-knee posterior back slab was applied postoperatively for soft-tissue protection and pain control. The postoperative course was uneventful, and the patient was discharged two days after surgery in good general condition (Figure 6).

Following surgery, the affected ankle was immobilized in a below-knee cast for the first two weeks to allow adequate soft-tissue healing and protect the surgical fixation. At the two-week postoperative follow-up, the cast was removed after satisfactory wound healing, and a supervised physiotherapy program was initiated.

During weeks two to four, the rehabilitation program consisted of active and passive ankle range of motion exercises, including dorsiflexion, plantarflexion, inversion, and eversion, together with triceps surae stretching, progressive calf muscle strengthening exercises, and proprioceptive and balance training, while maintaining a non-weight-bearing status to protect fracture healing and the repaired syndesmosis.

During weeks four to six, the same rehabilitation protocol was continued with progressive improvement of ankle mobility, muscle strength, and neuromuscular control. Partial weight bearing was then initiated as tolerated under the supervision of the treating physiotherapist.

From week six onward, the patient progressed to weight bearing as tolerated, while continuing ankle range of motion exercises in all planes, progressive calf muscle strengthening, triceps surae stretching, proprioceptive and balance training, and gait retraining until full functional recovery was achieved.

**Figure 7:**
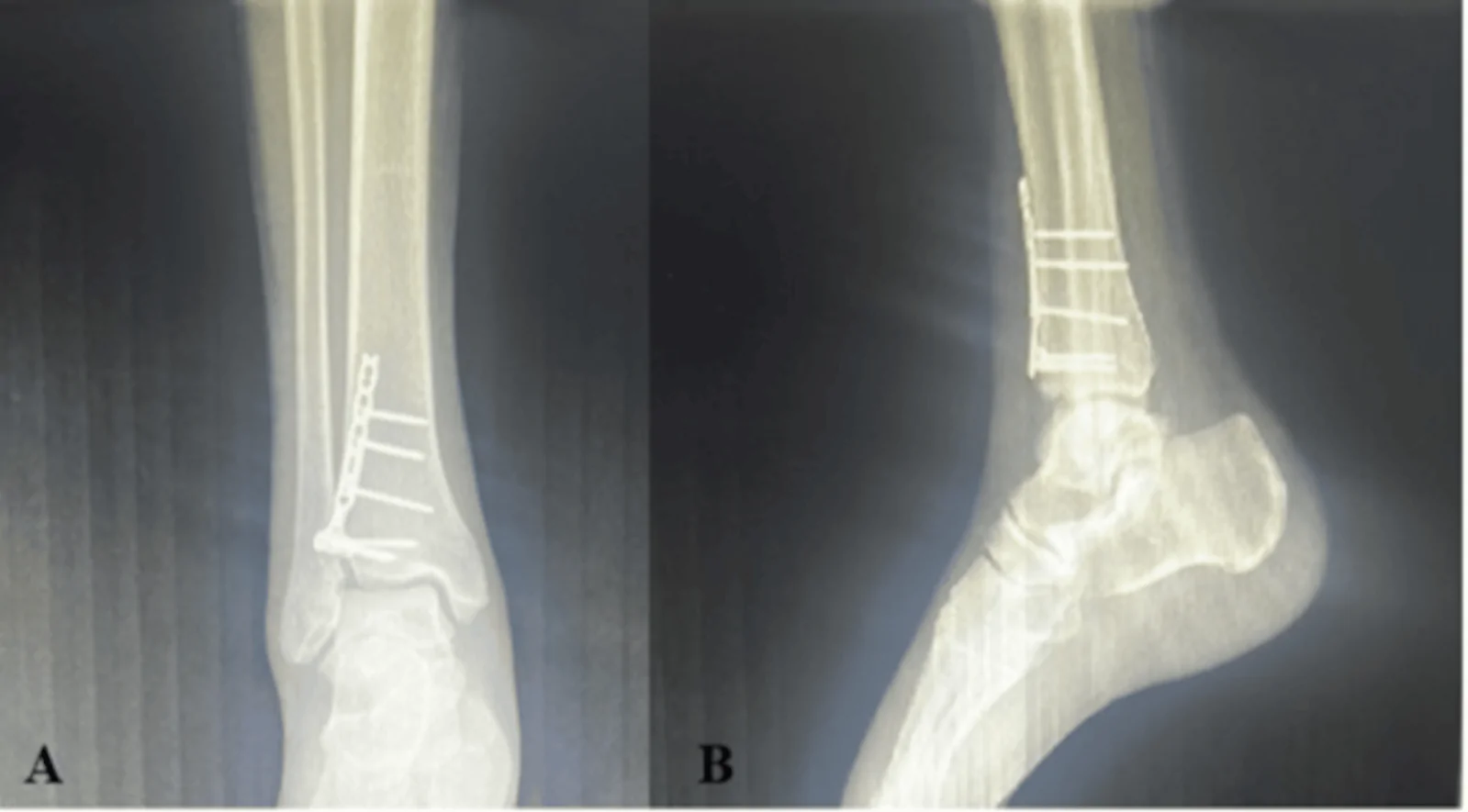
Follow-up X-rays. Three-month follow-up ankle anteroposterior (A) and lateral (B) X-ray views showing complete healing of the fracture without further displacement or disruption of the joint congruency.

The patient was followed for three months, during which complete fracture union was achieved (Figure 7). At the final follow-up, he had regained full, pain-free ankle and subtalar range of motion, with no residual pain, stiffness, or functional limitations, and had returned to his pre-injury level of daily activities without the need for assistive devices.

## Discussion

The ankle joint complex maintains stability during weight-bearing and rotational movements through the coordinated function of its osseous, ligamentous, and muscular stabilizers. Among these structures, the distal tibiofibular syndesmosis, particularly the AITFL, plays a pivotal role in preserving ankle stability and optimizing functional outcomes following injury [11]. Avulsion fractures at the tibial attachment of the AITFL, known as Chaput fractures, commonly result from external rotational forces and may compromise syndesmotic stability if left untreated. Because these fractures are often subtle and obscured by overlapping anatomical structures on conventional radiographs, accurate diagnosis may be challenging, and advanced imaging is frequently required to define the injury pattern and guide treatment [12].

The degree of syndesmotic instability depends not only on the avulsion fragment itself but also on the integrity of adjacent stabilizing structures, including the PITFL and the interosseous ligament. Partial syndesmotic injuries may be particularly difficult to recognize because both conventional imaging and intraoperative assessment can underestimate subtle instability [13]. Failure to identify and appropriately manage these injuries may result in chronic ankle instability, persistent pain, and post-traumatic degenerative changes. Consequently, restoration of syndesmotic stability through appropriate fixation is essential for achieving favorable long-term outcomes [14].

CT has become an indispensable adjunct in the evaluation of anterior malleolar injuries. Compared with plain radiographs, CT demonstrates a substantially higher sensitivity for detecting Chaput fractures, particularly small avulsion fragments, while providing superior assessment of fracture morphology, displacement, articular involvement, and fragment configuration [15]. These factors are critical for preoperative planning because treatment decisions are influenced more by fracture displacement and articular incongruity than by fragment size alone. In the present case, although initial radiographs suggested a Chaput fracture, CT imaging clearly demonstrated an isolated displaced fracture with an intra-articular step-off greater than 2 mm, confirming the indication for operative intervention.

Anterior malleolar fractures have been classified by Rammelt et al. into three distinct patterns: avulsion-type injuries, fractures involving the tibial incisura and articular surface, and impaction-related fractures [10]. Our patient sustained a Rammelt type III fracture, characterized by displacement involving the tibial incisura and articular surface. Because residual articular incongruity has been associated with inferior long-term outcomes, displaced fractures with an articular step-off exceeding 2 mm are generally considered indications for anatomical reduction and internal fixation to restore joint congruity, syndesmotic stability, and reduce the risk of post-traumatic arthritis [10].

The role of additional syndesmotic fixation after fixation of a Chaput fragment remains controversial. Several studies have reported comparable long-term functional and radiological outcomes in patients treated with and without syndesmotic fixation when anatomical reduction of the avulsion fragment successfully restores syndesmotic stability [16]. Dynamic fixation has been proposed as an alternative to static screw fixation because it permits physiological micromotion, facilitates earlier weight-bearing, and may reduce implant-related complications and the need for routine hardware removal [17]. Nevertheless, the indication for syndesmotic fixation should be individualized according to the residual stability of the syndesmosis after fracture reduction rather than applied routinely. Previous studies have also demonstrated that selected rotational ankle fractures with residual syndesmotic laxity may achieve satisfactory functional and radiological outcomes without syndesmotic fixation [18].

Restoring joint congruity and maintaining distal tibiofibular stability remain the primary objectives in the management of ankle fractures associated with syndesmotic injury. Although several fixation techniques have demonstrated satisfactory clinical outcomes, there remains no universal consensus regarding the optimal postoperative management, including the timing of weight-bearing and the routine removal of syndesmotic implants [19]. Comparative studies have likewise demonstrated no significant differences in clinical outcomes between dynamic and static syndesmotic fixation, although dynamic fixation may allow earlier mobilization while avoiding routine implant removal and preserving physiological syndesmotic motion [20].

In the present case, open reduction and internal fixation achieved anatomical reduction of the displaced Chaput fragment, and intraoperative fluoroscopic stress testing using both the external rotation stress test and Cotton test demonstrated restoration of syndesmotic stability without residual tibiofibular diastasis following anatomical reduction and fixation of the Chaput fragment. Consequently, additional syndesmotic fixation was not required. The patient demonstrated an excellent short-term clinical outcome, achieving complete fracture union, full pain-free ankle and subtalar range of motion, restoration of syndesmotic stability confirmed by clinical assessment, unrestricted full weight bearing, and return to pre-injury daily activities without residual pain or hardware-related complications at the three-month follow-up. This case further supports the importance of maintaining a high index of suspicion for isolated Chaput fractures, utilizing CT imaging for accurate diagnosis and surgical planning, and performing anatomical reduction in displaced fractures to restore syndesmotic stability while avoiding unnecessary syndesmotic fixation when intraoperative stability has been adequately restored.

## Conclusions

Isolated displaced Chaput fractures are an uncommon but clinically important manifestation of syndesmotic ankle injuries that may be overlooked on conventional radiographs. CT plays a pivotal role in accurately characterizing fracture displacement and articular involvement, thereby facilitating appropriate surgical planning. In the present case, anatomical reduction and stable internal fixation restored syndesmotic stability without the need for additional syndesmotic fixation, resulting in complete fracture union and an excellent clinical outcome at the three-month follow-up. Although this case supports the concept that fixation of the avulsed Chaput fragment alone may be sufficient to restore syndesmotic stability in carefully selected patients, treatment decisions should remain individualized and guided by meticulous preoperative imaging, intraoperative assessment of syndesmotic stability, and fracture morphology. Further studies with larger patient cohorts and longer follow-up are warranted to validate these findings and establish evidence-based treatment recommendations.
